# Supplementary material for: Phase transition in the cuprates from a magnetic-field-free stiffness meter viewpoint
Source: Nat Commun. 2019 Jun 5;10:2463. doi: 10.1038/s41467-019-10480-x (PMC6549142; doi:10.1038/s41467-019-10480-x)
Supplement: Supplementary file 1 — Supplementary Information [file 41467_2019_10480_MOESM1_ESM.pdf]

Phase transition in the cuprates from a magnetic-field-free stiffness meter viewpoint
Supplementary Information

Kapon et al.

(Dated: May 6, 2019)

SUPPLEMENTARY FIGURE 1

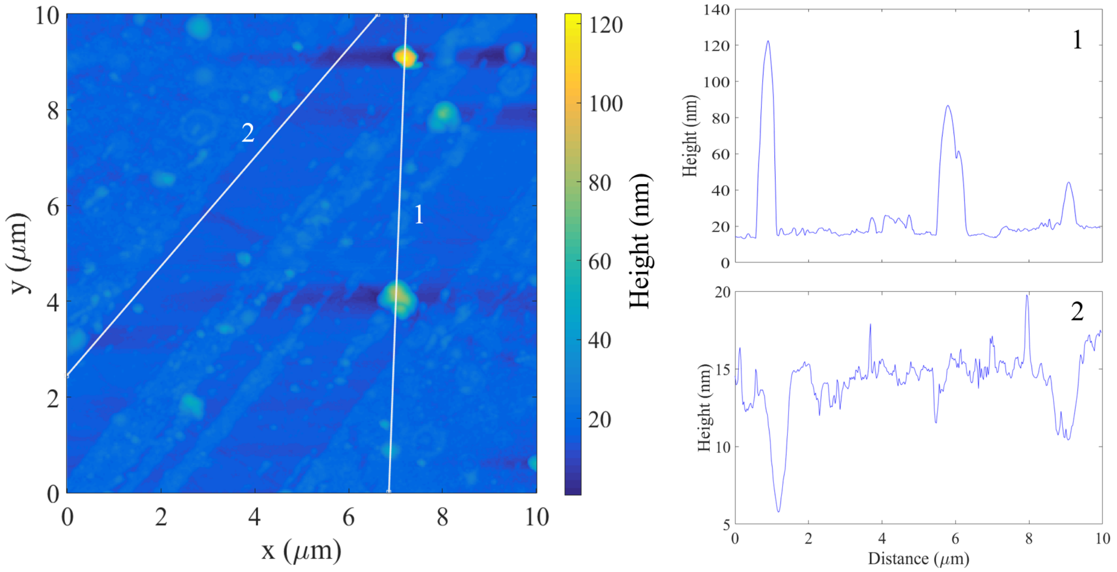

Supplementary Figure 1. **Sample surface roughness.** AFM image of one polished LSCO x=0.125 plate treated with 20 nm Alumina suspension. Height profiles along two lines are presented, demonstrating fairly smooth surface.

SUPPLEMENTARY NOTE 1: STIFFNESSOMETER

Zero External Field

There is a risk that field generated in the inner-coil leaks since no coil is infinitely long or perfect. To overcome this leak, a main coil, also shown in Fig. 2 in the manuscript, acts as a shim to cancel the field on the ring when it is at the gradiometer center. Our main-coil has a field resolution of 10^{-3} Oe from 0 up to 200 Oe. Therefore, we can keep the field on the ring as low as 1 mOe.

To ensure that our signal is not due to residual field, we measure the stiffness (i.e. zero external field and only applied current in the inner-coil) of closed and open rings, which are otherwise identical in size. The results are shown in Supplementary Fig. 2(a). The signal from a closed ring is much bigger than the background from an open one. In Supplementary Fig. 2(b) we repeat this measurement with an applied field in the main coil of 1 Oe, and no current in the inner-coil. In this case both open and closed rings give strong and similar signal. The difference between the two signals is consistent with the missing mass in the open ring. This test confirms that field leakage is not relevant to our stiffness measurement.

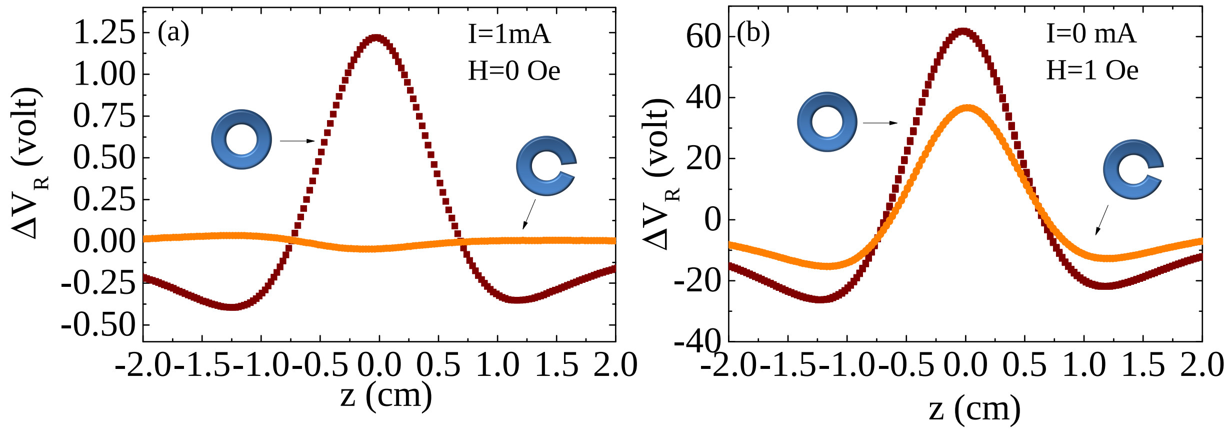

Supplementary Figure 2. **Experimental tests.** The SQUID signal for an open and closed rings when (a) the external field is zero and the curret in the inner coil is finite (finite vector potential), (b) when the inner coil current is zero but the external field is finite. The data prove that the magnetic field in the ring position is negligible.

Finite Coil

Supplementary Fig. 3 presents the vector potential of a finite coil as a function of the distance from its center, r, in mm, for different z positions. The coil is 60 mm high, has 0.54 mm polyamide core and two layers of windings with a 0.05 mm diameter wire. For all relevant z values, the potential drops off like $1/r$, as in the case of infinite coil, which appears in the figure in blue line. The different prefactor does not affect the Stiffnessometer PDE and its solution, as they are normalized by $A_{IC}(R_{PL})$, and the absolute value of the vector potential is measured by ΔV_{IC}^{max}.

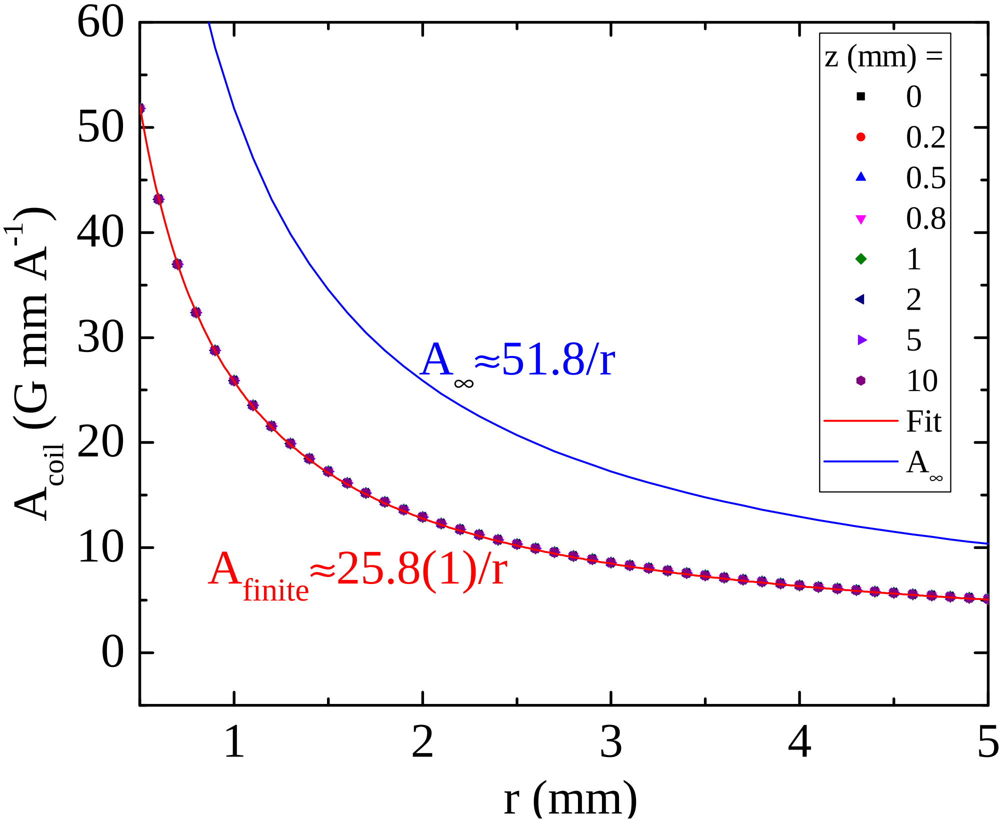

Supplementary Figure 3. **Finite versus infinte coil vector potential.** The potenetial of finite coil is presented for several z values. It falls off like $1/r$ and is z-independent inside the ring and outside of it at least until $z = 10$ mm. Blue line shows the potential of an infinite coil. Red line is a fit to the data.

Validity of London's Equation

In the Ginzburg-Landau theory, $\mathbf{J} = \rho_s \left(\hbar c/q \nabla \varphi - \mathbf{A} \right)$ where φ is the phase of the order parameter and q is the carriers charge. However, as explained in the main text, the procedure of cooling in zero vector potential and turning it on only at base temperature, sets $\nabla \varphi = 0$, and consequently $\mathbf{J} = -\overline{\rho}_s \mathbf{A}$. To see this one can view the phase φ as an in-plane arrow. Cooling at $\mathbf{A} = 0$ must set $\nabla \varphi = 0$ to minimize the kinetic energy, namely, all the arrows point in the same direction. Since the phase is quantized, to change φ means to make a twist of all arrows in a closed loop, such that the phase between the first arrow and last one in the loop changes by 2π. This would lead to a discontinuity in the phase value, a procedure that costs energy. A nice analog is a ferromagnetic ring with the spins pointing in the same direction. Rotating the last spin with respect to the first one by 2π requires to break a bond. This procedure is not energetically favorable for a ferromagnet (or the SC ring). Therefore, when turning $\mathbf{A}$ on after cooling, all the arrows continue to point in the same direction and $\nabla \varphi = 0$, until A exceeds a critical value. At this point, the current in the SC is too high and it is worthwhile for the superconductor to "break a bond" and reduce the current.

To prove London's proportionality in our system, we measure ΔV_R^{max} as a function of the current in the inner-coil at constant temperature. This is depicted in the inset of Fig. 3(a) in the main text for LSCO c-ring at $T = 29.92(5)$ K and for the a-ring at $T = 29.28(5)$. The signal from the ring is proportional to the applied current in the inner-coil until ΔV_R^{max} reaches a saturation value. It means that the superconductor can generate only a finite amount of current. The critical vector potential allows one to determine the coherence length ξ. Using relation 4.37 from [1], Supplementary Fig. 3, and the critical current presented in Fig. 3(a) we estimate $\xi \approx 50$ nm. A more accurate determination of ξ is given in Ref. [2]. We find that $\xi \ll \lambda$ at least up to $T = 29.92(5)$ K.

SUPPLEMENTARY NOTE 2: NUMERICAL METHODS

Here we provide more details about the numeric solution of Eq. 3 in the anisotropic case. The gauge choices are as follows: Inside the ring, applying divergence to Eq. 3 yields the gauge $\nabla \cdot (\overline{\rho}_s \mathbf{A}_{tot}) = 0$, where $\mathbf{A}_{tot} = \mathbf{A_R} + \mathbf{A_{IC}}$. This gauge also enforces the continuity equation for the current density $\mathbf{J} = \overline{\rho}_s \mathbf{A}_{tot}$. Outside the ring we apply the Coulomb gauge $\nabla \cdot \mathbf{A}_{tot} = 0$, which is also used to determine $\mathbf{A_{IC}}$ and $\mathbf{A}_{tot}$ in the isotropic case. The boundary conditions are $A(\infty) = 0$. In practice, infinity is understood as the domain surface, and the domain is taken to be large enough so that finite-domain effects are negligible. The domain of the problem is defined as a cylinder with height 100 times that of the ring, i.e. $7.7R_{PL}$ and outer radius 100 times that of the ring, i.e. $11.5R_{PL}$. Since no current can cross the ring surface, we demand $\mathbf{J}_\perp(\mathbf{r}_{in}) = \mathbf{J}_\perp(\mathbf{r}_{out}) = 0$ where $\perp$ stands for the direction perpendicular to the surface, and r_{in} (r_{out}) is the inner (outer) radius of the ring. Finally, from the absence of a surface field, we demand $\Delta\mathbf{A}_\parallel(\mathbf{r}_{in}) = \Delta\mathbf{A}_\parallel(\mathbf{r}_{out}) = 0$, where $\Delta\mathbf{A}_\parallel$ stands for the difference between the vector potential parallel to the surface inside the ring and outside of it.

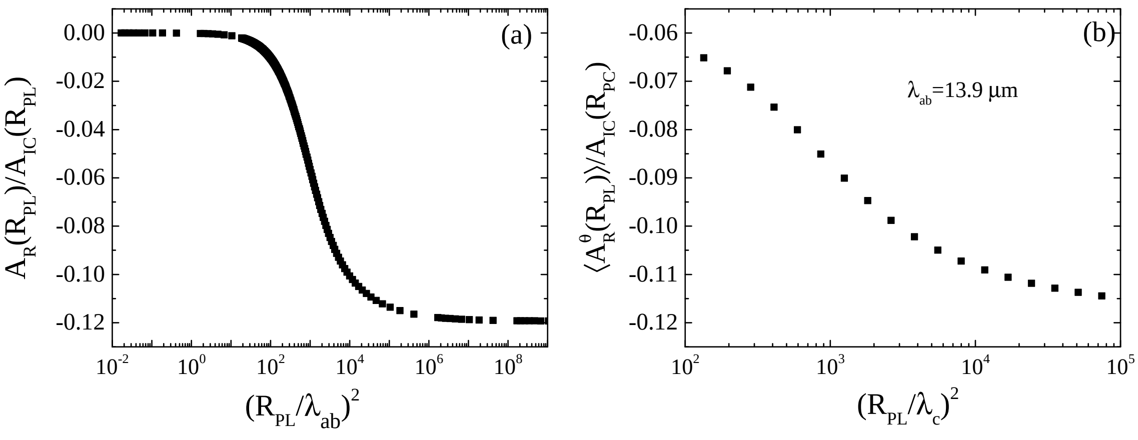

Supplementary Figure 4. **Extracting the stiffness.** Numerical results of the vector potentials ratio as appears in Eq. 2 as a function of (a) $(R/\lambda_{ab})^2$ and (b) $(R/\lambda_c)^2$ for $\lambda_{ab} = 13.9$ μm at $T = 29.16$ K.

Supplementary Fig. 4 shows the numerical results of the vector potentials ratio that appears in Eq. 2 as a function of (a) $(R/\lambda_{ab})^2$ and (b) $(R/\lambda_c)^2$ for $\lambda_{ab} = 13.9$ μm at $T = 29.16$ K. In our analysis, λ_{ab} is extracted from the c-ring data in the isotropic case. Then, for each temperature, the corresponding λ_{ab} is used to generate the result in panel

(b), and combining with the a-ring data λ_c is extracted.

Supplementary Fig. 5 presents the numeric solution of the ring vector potential A_R at $z = 0$ plane (midheight of the ring), calculated for LSCO x=0.125 a-ring at $T = 29.16$ K with $\lambda_{ab} = 13.9$ μm, extracted from the extrapolation function presented in the main text, and $\lambda_c = 145$ μm. Panel (a) shows the azimuthal part of $\mathbf{A}$, whereas panel (b) the radial one.

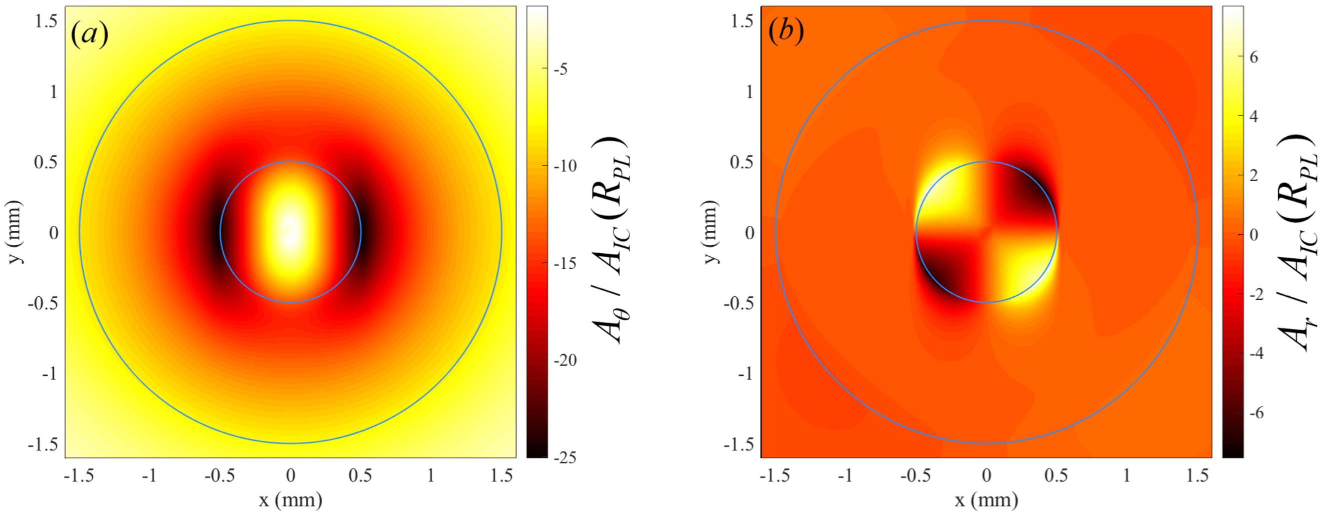

Supplementary Figure 5. **Vector potential for LSCO x=0.125 a-ring.** Numeric solution of the radial (a) and azimuthal (b) components of the vector potential inside the ring at z=0 for $\lambda_{ab} = 13.9$ μm and $\lambda_c = 145$ μm

Supplementary Fig. 6 shows the absolute value of the current density $\mathbf{J}$ inside the rings for two cuts at fixed angeles: (a) xz plane, (b) yz plane. At the xz plane, the current concentrates at a very thin layer close to the ring inner rim, while in the yz plane the current penetrates further into the bulk. This corresponds, of course, to the large difference in the penetration depth in the two directions.

Finally, Supplementary Fig. 7 shows the magnetic field generated by the ring as calculated from the curl of $\mathbf{A_R}$. The penetration pattern of the field is of an ellipse due to the penetration depths anisotropy.

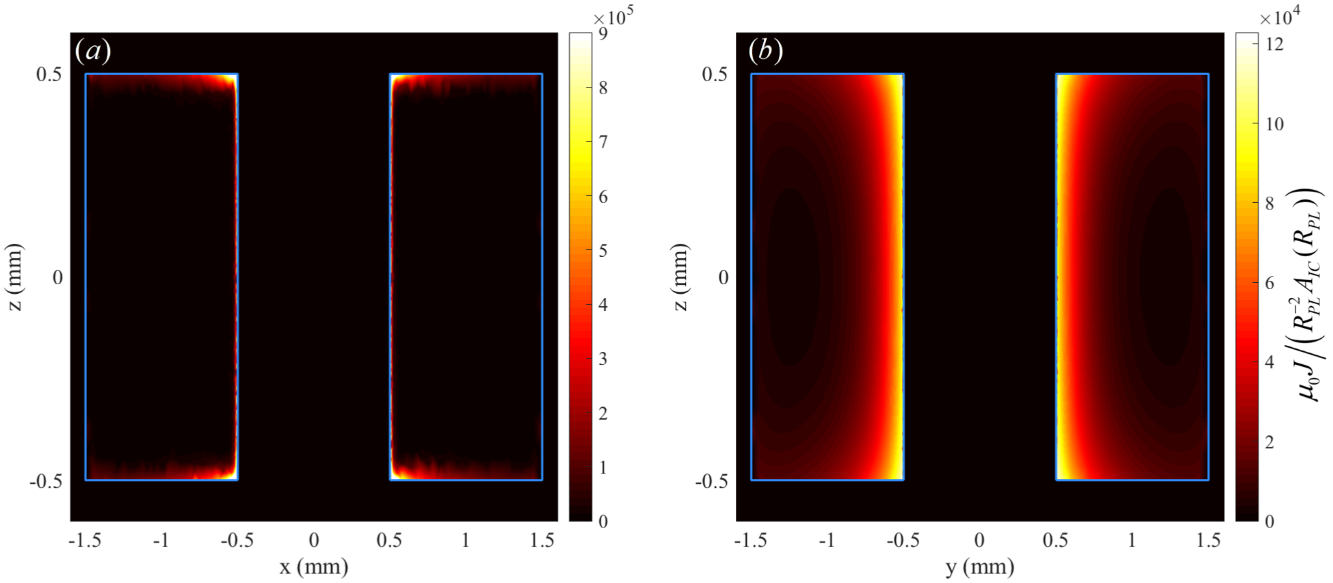

Supplementary Figure 6. **Current density simulation inside LSCO x=0.125 a-ring.** False color map of the current density distribution in a ring with $\lambda_{ab} = 13.9\ \mu m$ and $\lambda_c = 145\ \mu m$ in the (a) xz plane and (b) yz plane. Most of the current concentrates on the inner rim.

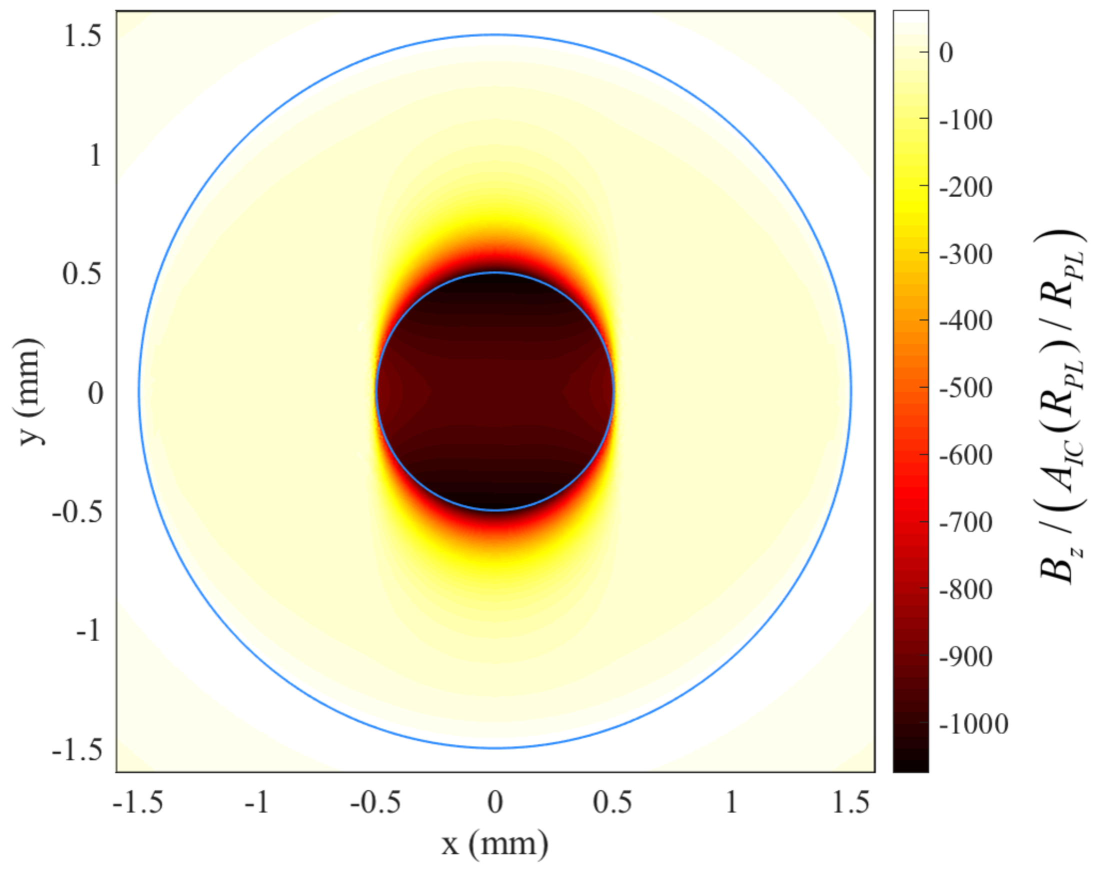

Supplementary Figure 7. **Magnetic field** False color map of the magentic field z component inside the a-ring and its vicinity for $\lambda_{ab} = 13.9\ \mu m$ and $\lambda_c = 145\ \mu m$

SUPPLEMENTARY NOTE 3: LE-μSR

There are two methods by which one can extract the penetration depth. The simple method is to fit each data set (at each temperature and energy) to $A(t) = A_0 \exp(-t/T_2) \cos(\omega t)$. From this fit one can extract asymmetry, relaxation, and the average internal field as a function of average implantation depth and temperature. Supplementary Fig. 8 summarizes the internal magnetic field as a function of implantation energy for different temperatures and field orientations. The field here is calculated by $B = \omega/2\pi\gamma$, where ω is the angular frequency of the muon polarization and γ is the gyromagnetic ratio. Noticeably, close to the surface and at low T, the magnetic field does not change with increasing implantation depth for $\mathbf{H} \parallel \mathbf{c}$. Only for energies above 5 keV does a linear trend of decay appears. This 10 to 20 nanometers of "dead layer" could be a byproduct of the polishing process.

Supplementary Fig. 9 depicts the temperature dependence of the individual fit parameters for the highest implantation energy. The magnetic field (panel (a)) seems to behave erratically close to the phase transition into the superconducting state. We attribute this behavior to demagnetization factor and mutual coupling between different pieces of the sample. The asymmetry (panel (b)) decreases upon cooling since LSCO x=0.125 is known to have a magnetic phase concomitant with the superconducting one [3–5]. The muon spin relaxation (panel (c)) has a peak at the critical temperature, which is also unusual.

The presence of magnetism could be detrimental to our analysis if it depends on depth. To verify that this is not the case, we perform zero field (ZF) measurements for different implantation energies at $T = 5$ K well below T_c and for $T = 30$ K above T_c. The results are presented in Supplementary Fig. 10. Fast relaxation and reduction of the asymmetry are observed at low temperature due to local random fields originating from the magnetic stripes in the sample. Nevertheless, there is no change in the magnetic relaxation with implantation depth.

The more sophisticated analysis method is presented in the main text. For each temperature, we fit all data sets with energy larger than 5 keV due to the presence of a dead layer, using Eq. 5. In the fit A_0 is a free parameter, and λ, u and B_0 are shared. A_0 is free because the number of muons actually penetrating the sample varies with energy. u represents relaxation processes that are implantation depth independent such as magnetism or field variations perpendicular to x. These are taken into account as some Lorentzian prob-

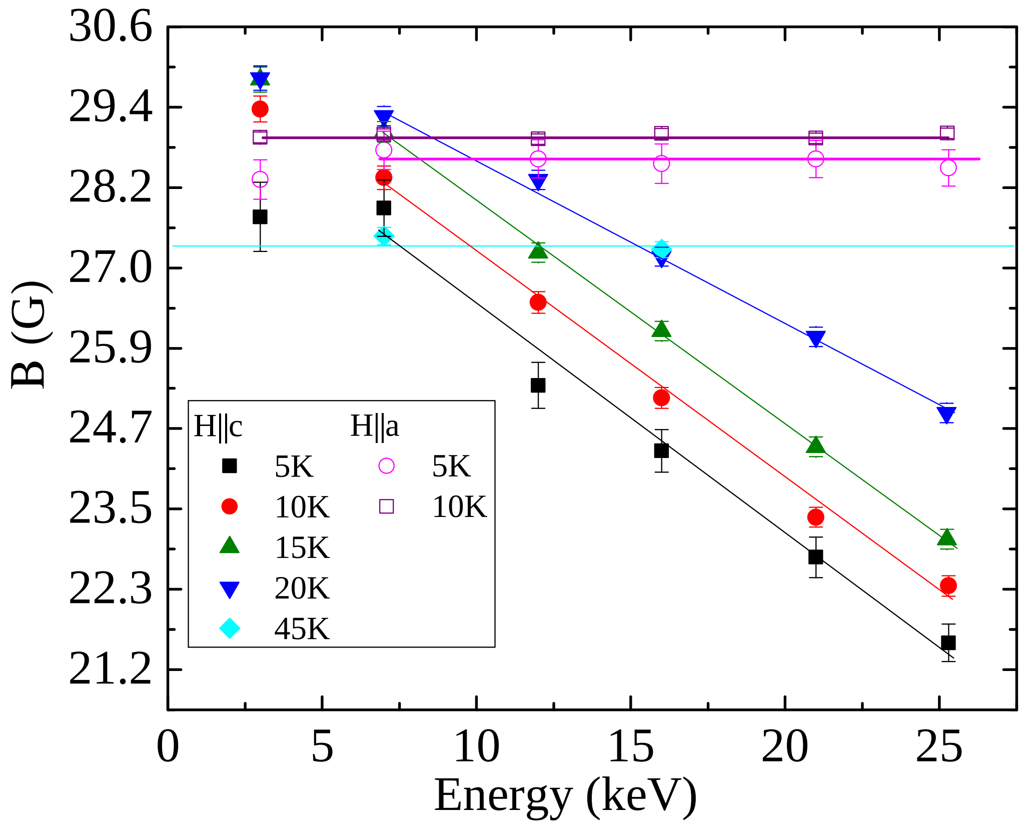

Supplementary Figure 8. **Magnetic field as function of implantation energy.** Closed symbols are $\mathbf{H} \parallel \mathbf{c}$ and open symbols are $\mathbf{H} \parallel \mathbf{a}$. Straight lines are guides to the eye. The magnetic field below $E = 5$ keV does not fit the linear trend of the field decay, indicating a dead layer of about 10 to 20 nanometers, possibly caused by the polishing treatment. Error bars are the results of the fitting procedure.

ability distribution of the total internal magnetic field with FWHM of $2/u$. λ and B_0 are naturally common to each temperature. Comparing the two analysis methods for $T = 25$ K, for example, the penetration depths agree within 20%.

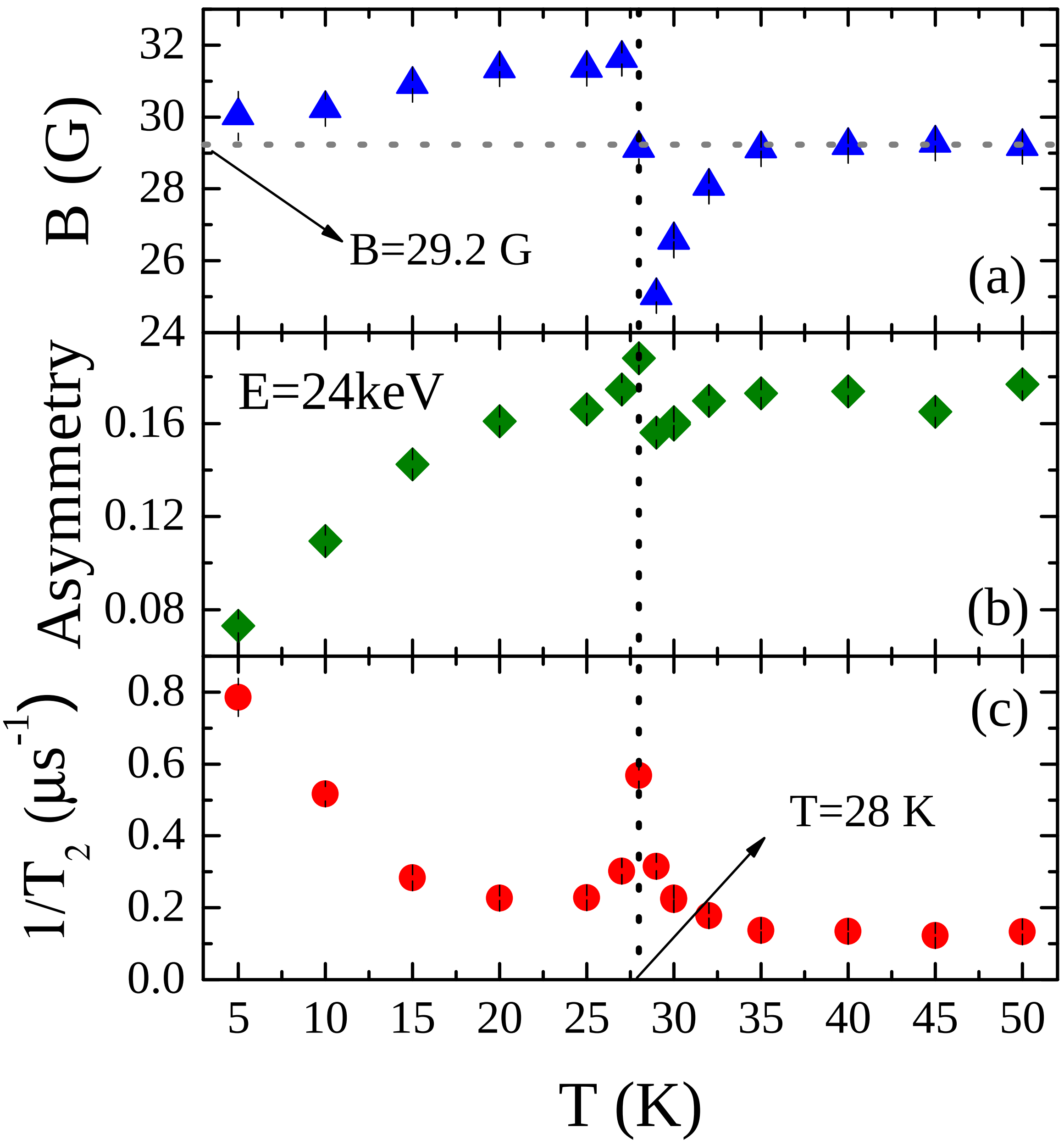

Supplementary Figure 9. **Temperature dependance of LE-μSR parameters**. The measurement was done at constant energy of 24 keV. The magnetic field (a) displays peculiar behavior near T_c. Its magnitude below T_c is larger than that of the normal state. The asymmetry (b) is constant until 20K, where it starts to drop due to magnetic freezing. The magnetism is also exhibited in an uprise of the decay rate (c) at low temperatures.

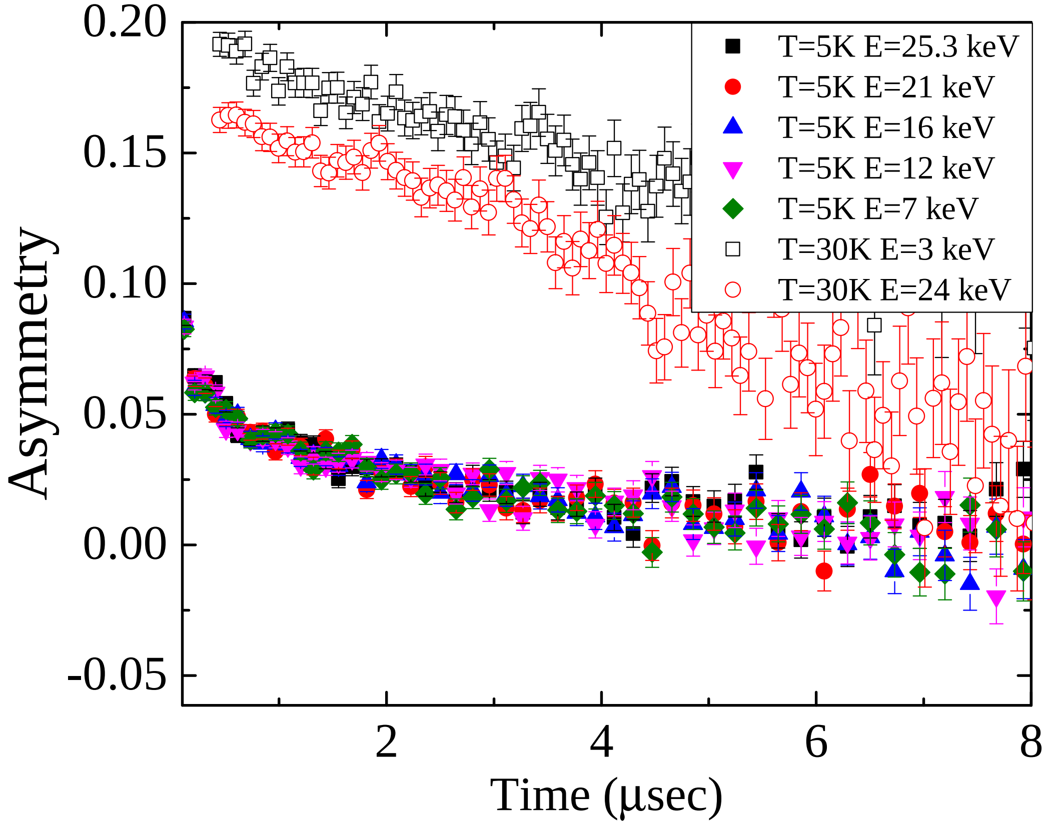

Supplementary Figure 10. **Depth independent magnetism in LSCO x=0.125**. Asymmetry vs. time at T = 5 K (close symbols) and T = 30 K (open symbols) for different implantation energies. The signal does not change as a function of energy at low temperatures, justifying a depth independent relaxation component (see main text).

[1] Tinkham, M. *Introduction to superconductivity* (Courier Corporation, 2004).

[2] Kapon, I., Golubkov, K., Gavish, N. & Keren, A. Stiffnessometer, a magnetic-field-free superconducting stiffness meter and its application. *arXiv preprint arXiv:1705.00624* (2017).

[3] Panagopoulos, C. *et al.* Evidence for a generic quantum transition in high-T_c cuprates. *Physical Review B* **66**, 064501 (2002).

[4] Suzuki, T. *et al.* Observation of modulated magnetic long-range order in $La_{1.88}Sr_{0.12}CuO_4$. *Phys. Rev. B* **57**, R3229–R3232 (1998).

[5] Kimura, H. *et al.* Neutron-scattering study of static antiferromagnetic correlations in $La_{2-x}Sr_xCu_{1-y}Zn_yO_4$. *Phys. Rev. B* **59**, 6517–6523 (1999).
